# Prevalence of Selected Immune Evasion Genes and Clonal Diversity in Methicillin-Susceptible *Staphylococcus aureus* Isolated from Nasal Carriers and Outpatients with Cut Wound Infections

**DOI:** 10.3390/antibiotics13080730

**Published:** 2024-08-03

**Authors:** Gabriela Jura, Helena Masiuk, Agata Pruss, Mateusz Kurzawski, Monika Sienkiewicz, Iwona Wojciechowska-Koszko, Paweł Kwiatkowski

**Affiliations:** 1 Department of Diagnostic Immunology, Pomeranian Medical University in Szczecin, 70-111 Szczecin, Poland; 2 Department of Medical Microbiology, Pomeranian Medical University in Szczecin, 70-111 Szczecin, Poland; 3 Department of Laboratory Medicine, Pomeranian Medical University in Szczecin, 70-111 Szczecin, Poland; 4 Laboratory of Pharmacodynamics, Pomeranian Medical University in Szczecin, 71-899 Szczecin, Poland; 5 Department of Pharmaceutical Microbiology and Microbiological Diagnostic, Medical University of Lodz, 90-151 Lodz, Poland

**Keywords:** *Staphylococcus aureus*, MSSA, outpatients, nasal carriers, wound infections, immune evasion, clonal diversity

## Abstract

*Staphylococcus aureus*, being one of the most common human pathogens, is responsible for infections in both hospital and community settings. Its virulence is attributed to its ability to evade the immune system by producing immune evasion (IE) proteins. The aim of this study was to detect the frequency of selected IE genes (*spin*, *sbi*, *sea*, *sak*, *chp*, *scin*, *sep*, *ecb*), belonging to the immune evasion cluster (IEC), and IEC types in 86 methicillin-susceptible *S. aureus* (MSSA) strains isolated from unrelated outpatients. In order to determine the diversity of analyzed strains, the phylogenetic relatedness was also determined. All strains were examined for the presence of IE genes using polymerase chain reaction assay. To analyze the clonal relatedness of *S. aureus*, pulsed-field gel electrophoresis (PFGE) was performed. All analyzed strains harbored the *scn* gene, followed by *sbi* (95.4%), *ecb* (91.7%), *spin* (89.5%), *sak* (83.7%), *chp* (67.4%), *sep* (67.4%) and *sea* (5.8%). Seventy-three (84.9%) *S. aureus* strains were classified into IEC types, of which, IEC type F was most commonly observed. IEC type A was not detected. PFGE results showed no association between clonal relatedness and the presence of IE genes/IEC types. In conclusion, the abundant and so diverse repertoire of genes determining invasion in analyzed strains may prove the fact that these strains are highly advanced and adapted to evade the host immune response.

## 1. Introduction

*Staphylococcus aureus* is a major human pathogen that is responsible for a wide range of infections in both hospital and community environments. *S. aureus* is considered the most common cause of skin and soft tissue infections (SSTIs), bacteremia, osteomyelitis, toxin-mediated food poisoning, and necrotizing pneumonia in both outpatients and inpatients [1]. It is estimated that approx. 30% of the human population is colonized by *S. aureus* in the nasal cavity [2]. Following the auto-transmission, bacteria spread to different body sites, leading to endogenous infections [3]. Overuse of antibiotics contributes to the rising trend of drug resistance among *S. aureus*, which has been demonstrated in recent years [4]. It is worth noting that the ability of this bacterium to cause the infection and to survive within the host is determined by its bacterial ability to evade the host’s immune responses, since the treatment management is frequently impaired by acquired antibiotic resistance to various groups of antimicrobial drugs, including beta-lactams, macrolides and fluoroquinolones [5].

The main immune defense mechanism against *S. aureus* involves the activation of phagocytic cells. The process, mediated primarily by neutrophils, leads to engulfment, inactivation and destruction of the bacteria [6]. *S. aureus* cells, in order to avoid this type of immune response, are increasingly producing more effective mechanisms of conditioning the exacerbation of infection symptoms [7]. The bacterium, in order to avoid the innate host immune mechanisms of responses, also “learns” how to become unrecognized.

The constant battle between the human body’s defense mechanisms and *S. aureus* is ruled by its ability to produce immune evasion (IE) proteins, such as staphylococcal peroxidase inhibitor (SPIN; encodes by *spin* gene and inhibits myeloperoxidase activity), staphylococcal immunoglobulin-binding protein (SBI; encoded by the *sbi* gene and binds to IgG), staphylococcal enterotoxin A (SEA; encoded by *spa* gene and stimulates T-cell proliferation), staphylokinase (SAK; encoded by *sak* gene and converts human plasminogen into plasmin), chemotaxis inhibitory protein of *S. aureus* (CHIPS; encoded by *chp* gene and interacts with complement 5a receptor [C5aR] and formyl peptide receptors [FPRs]), staphylococcal complement inhibitor (SCIN; encoded by *scn* gene and interacts with the C3b and Bb components), staphylococcal enterotoxin P (SEP; encoded by *sep* gene and stimulates T-cell proliferation) and extracellular complement-binding protein (ECB; encoded by *ecb* gene and inhibits the complement system by binding to the C3d). It is worth emphasizing that *sea*, *sak*, *chp*, *scn* and *sep* genes are included in the *S. aureus* IE cluster (IEC), classified into eight types: A (*sea*-*sak*-*chp*-*scn*), B (*sak*-*chp*-*scn*), C (*chp*-*scn*), D (*sea*-*sak*-*scn*), E (*sak*-*scn*), F (*sep*-*sak*-*chp*-*scn*), type G (*sep*-*sak*-*scn*) and H (*scn*) [6,8,9,10].

Methicillin-susceptible *S. aureus* (MSSA) strains analyzed by us to date have shown a highly variable repertoire of virulence genes, while the proportion of methicillin-resistant *S. aureus* (MRSA) strains isolated from out-of-hospital patients at our institution has remained stable over the years [11]. Most studies carried out worldwide focus primarily on MRSA strains, which are considered to have a high epidemic potential, whereas strains from infections, as well as from colonization, show, in majority, susceptibility to methicillin and are more genetically diverse depending on the type of SSTIs, as shown by pulsed-field gel electrophoresis (PFGE) analysis [3,11,12,13]. According to the available literature, PFGE is considered as the “gold standard” in epidemiological investigations [14].

Evaluation of the presence of evasion genes in *S. aureus* isolated from cut wounds, as well as from healthy nasal carriers, may shed clearer light on the true capacity of these strains and their ability to coexist with the host without causing infection. The therapeutic challenge faced by modern dermatology is to develop effective strategies for the treatment of SSTIs in ambulatory patients for whom only the topically or orally applied antibiotics are available, since the Polish recommendations regarding the screening of *S. aureus* carriers in order to assess the risk of endogenous infection apply only to patients prior to planned cardiac surgery [15]. Determination of *S. aureus* carriage in other patients is discretionary and performed only upon physician’s request. In the present study, the selection of study groups of *S. aureus* strains was not coincidental. We were specifically interested in the variations in the lack of *S. aureus* in the nasal vestibule of patients with a cut wound infection. The mere fact of infection, which might not result from auto-transmission, encouraged us to compare the genetic profiles of strains isolated from these patients with strains from healthy carriers.

By successively characterizing *S. aureus* isolated from ambulatory patients, we would like to evaluate the possible importance of simultaneous determination of *S. aureus* nasal carriage in order to reduce the risk of auto-transmission and the occurrence of staphylococcal infections. Knowing the genetic background of both groups of strains and their ability to evade the host immune response is also a prerequisite for us to plan further analyses of the serum antibody profile against individual virulence factors, both in patients with SSTIs and in healthy carriers.

Due to implementation of an appropriate antibiotic policy and sanitary regime, *S. aureus* has a low ranking among hospital-acquired strains isolated from Polish patients, while SSTIs in community settings caused by *S. aureus* are among the most common. The majority of the scientific data focus primarily on MRSA strains isolated from hospital outbreaks and strains found in outpatient settings presenting increasing antimicrobial resistance [16,17,18,19,20]. In contrast, our analyses to date indicate that *S. aureus* isolated from outpatients, despite being susceptible to most of the drugs, has a very diverse virulence gene repertoire [11]. We consider it worthwhile to understand the genetic structure of the different *S. aureus* groups and to determine their possible associations with particular infections.

Hence, the study aimed to determine the prevalence of selected IE genes (*spin*, *sbi*, *sea*, *sak*, *chp*, *scin*, *sep*, *ecb*), belonging to IECs, and IEC types in MSSA strains isolated from the nasal cavity of healthy individuals and from outpatients with cut wound infections. Special attention was paid to reveal the phylogenetic relatedness in order to determine the genetic diversity of the analyzed strains.

## 2. Results

### 2.1. PCR Results

The presence of IE genes was confirmed in both reference and analyzed strains (Table 1 and Supplementary Table S1). In all isolates, the presence of at least one IE gene was confirmed. The most frequently detected IE gene was the *scn* (*n* = 86; 100%), followed by *sbi* (*n* = 82; 95.4%), *ecb* (*n* = 79; 91.7%), *spin* (*n* = 77; 89.5%), *sak* (*n* = 72; 83.7%), *chp* (*n* = 58; 67.4%), *sep* (*n* = 58; 67.4%) and *sea* (*n* = 5; 5.8%) genes. Interestingly, only the *ecb* gene was significantly more frequently observed in strains isolated from nasal carriers than in strains isolated from patients with cut wound infections (*p* = 0.0058).

Results of the present study showed that seventy-three (84.9%) *S. aureus* strains were classified into IEC types. IEC type F was the most commonly observed in all analyzed strains (*n* = 43; 50.0%), whereas IEC type A was not confirmed. The prevalence of the other IEC types ranged from 1.2% (*n* = 1) to 24.4% (*n* = 21). Interestingly, non-typeable IEC variants (harboring all IEC genes) were statistically more frequently found in strains isolated from cut wounds (*p* = 0.0067) than in *S. aureus* isolated from nasal carriers. Detailed data regarding the number and prevalence of IEC types are listed in Table 2 and Supplementary Table S1.

### 2.2. PFGE Results

**Figure 1:**
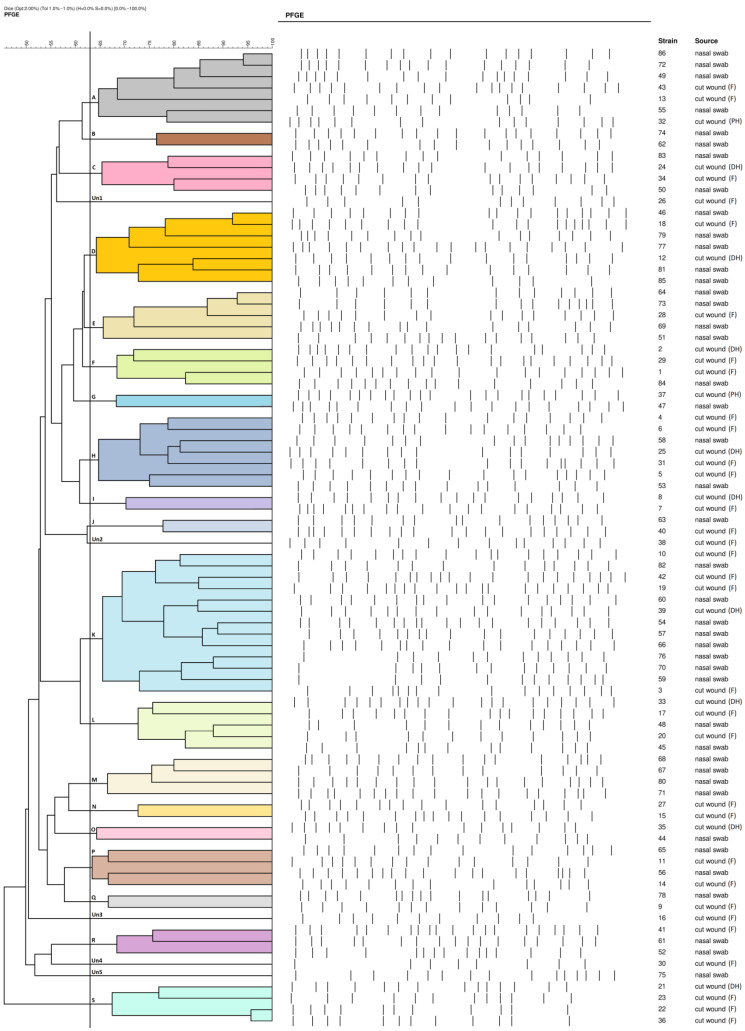
Dendrogram presenting pulsed-field gel electrophoresis (PFGE) profiles of 86 methicillin-susceptible *S. aureus* isolated from nasal carriers and outpatients with cut wound infections (DH—dorsal hand wound infection; F—finger infection; PH—palmary hand wound infection). PFGE profiles were clustered into 19 genotypes (A to S) and 5 unique (Un) patterns (Un1 to Un5), on the basis of a similarity threshold ≥63%.

Based on the performed study, 19 genotypes and five unique patterns were distinguished (Figure 1, Supplementary Table S1 and Supplementary Figure S1). Genetic type K was the most prevalent and was represented by 13 *S. aureus* strains. The other PFGE types were represented by a various number of isolates (from two to seven isolates).

**Figure 2:**
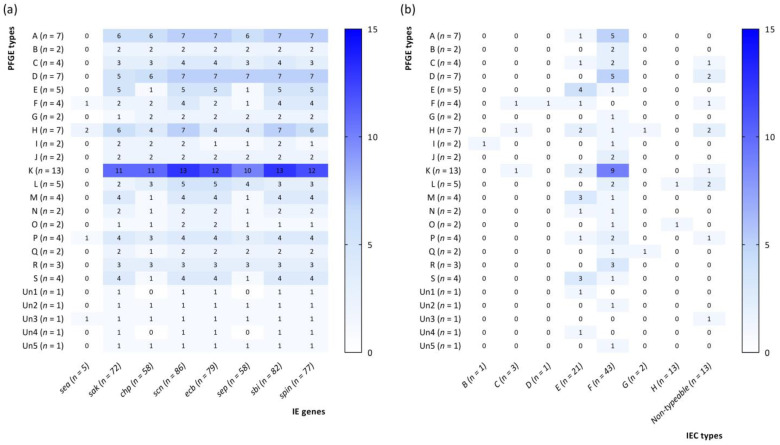
Correlation between pulsed-field gel electrophoresis (PFGE) profiles and the number of methicillin-susceptible *Staphylococcus aureus* strains harboring immune evasion (IE) genes (**a**) or immune evasion cluster (IEC) types (**b**).

After examining the strains, the authors observed there was no relationship between the PFGE type and the presence of IE genes/IEC types. Interestingly, *S. aureus* strains possessing IEC type F were most represented by genetic type K; however, these results were statistically insignificant. Detailed data on the correlation between the number of strains harboring IE genes or possessing IEC types and PFGE profiles are presented in Figure 2.

## 3. Discussion

The authors of this study performed a genetic analysis to detect selected IE genes and identify IEC types in clinical MSSA strains isolated from carriers and patients with cut wound infections. Of 86 strains of *S. aureus*, 84.9% were classified into IEC types. This result is similar to that obtained by Verkaik et al. [21] and van Wamel et al. [22], who determined the presence of IEC in about 90.0% of strains isolated from clinical *S. aureus* strains. IEC cluster typing in all the examined strains revealed the presence of the particular types with different ratios within both examined groups of strains. The presence of IEC from B to H cluster-type genes with the lack of A cluster-type genes was determined in all strains regardless of the place of collection. It is worth noting that non-typeable strains, which were also detected, were found statistically more frequently in strains isolated from cut wound infections. The presence of IEC cluster genes in variable compositions may be related to the spreading dynamics of various bacteriophages encoding particular IEC types [21]. According to available studies, IEC genes are much more frequently found in MSSA than in MRSA strains, which corresponds to the results obtained in the present study. According to van Wamel et al. [22], nearly 90% of human MSSA isolated from various infections harbored IEC-carrying βC-Φs prophage, whereas Ahmadrajabi et al. [23] confirmed the absence of IEC in most of hospital-acquired methicillin-resistant *S. aureus* (HA-MRSA) strains. Nevertheless, different results were obtained by Kmiha et al. [24], who showed the presence of IEC in almost 95% of MRSA strains isolated from burn wound infections. These results may support the observation of the autonomous ways of acquiring resistance and IEC genes. Moreover, it may allow for initial differentiation of the origin of strains derived from hospitalized or community patients, since community *S. aureus* is most commonly the cause of SSTIs [25,26,27].

In the present study, IEC types F and E were characterized with the highest prevalence rates, which were 50.0% and 24.42%, respectively. These values are in contrast to the results of studies obtained by Baptista et al. [10], Chai et al. [28] and Bano et al. [29], who determined the absence of IEC type F in *S. aureus* isolated from food handlers’ hands and nostrils [10], animal handlers (nasal and oral swabs) [28] and from cancer patients (wound and throat swabs, skin lesions, ear fluid) [29]. Other studies revealed that IEC type F occurred less frequently than in the present study, where the incidence rates were 4.4% (for strains isolated from blood, joint, pericardial dialysis, pulmonary fluid and liquor) and 37.0% (for strains isolated from swine and from humans with long-term and short-term contact with swine) [22,30]. Furthermore, the absence of IEC types A and B in one strain constitutes a significant difference between results obtained in the present study and available data. Baptista et al. [10] observed that IEC types A and B were most prevalent as they were detected in 40.0% of strains isolated from food handlers’ hands and nostrils. In total, 21.4% of type A and 15.5% of type B were noted in strains isolated from cancer and non-cancer patients (wound and throat swabs, skin lesions, ear fluid), respectively [29]; 12.2% of type A and 26.7% of type B were observed in *S. aureus* clinical strains (blood, joint, pericardial dialysis, pulmonary fluid and liquor), respectively [22]; 49.3% of type B genes were noted in swine [30]; and, finally, 58.0% of type B genes were isolated from persistent carriers (serial nasal swab) [21].

In the current study, the most frequently detected IEC genes were the *scn* (100%), followed by *sbi* (95.4%), *ecb* (91.7%), *spin* (89.5%), *sak* (83.7%), *chp* (67.4%), *sep* (67.4%) and *sea* (5.8%). According to available studies, SCIN and CHIPS proteins are secreted most abundantly during the *S. aureus* exponential growth phase, during early stages of infection. SCIN and CHIPS inhibit opsonization, chemotaxis and phagocytosis, acting together as a potent blocking system of immune responses in early inflammatory stages [31]. Though inflammation is a body’s natural response to pathogens in either acute or chronic wounds, their clinical presentation is significantly different, i.e., it is noticeable clinically in acute wounds, but much less in chronic wounds. Initial exposure of *S. aureus* to host tissues, such as mucosal surfaces or skin, is thought to activate the process of phagocytosis to kill the pathogen, which, by producing virulence genes, increases the host’s immune response [32]. This is a crucial point for *S. aureus* to inactivate the first line of host defense [33].

In both groups of patients, genes encoding SCIN, SAK, SBI and SPIN were detected in a similar number of strains. Worth underlying is the fact that no SSTIs episodes were reported within the group of nasal carriers. Thus, it is essential to understand host adaptive immune responses which allows *S. aureus* to contribute to the carrier state without triggering inflammation, which is linked with individual immune response variability in carriers [34].

It is estimated that *S. aureus* is capable of producing more than 200 evasion proteins, but the detailed mechanisms of action for many of these have not yet been fully understood [35,36]. IE proteins (including those encoded by the IEC system) are the most important *S. aureus* virulence factors responsible for evading the host immune system, providing bacterial survival [37]. One of the major host defense mechanisms against *S. aureus* producing IE is inhibition of alternative pathways of the complement (C). C3a and C5a protein subunits condition the process of phagocytosis of bacterial cells [38]. *S. aureus* evasion proteins specialize in avoiding and inhibiting the complement system by binding C3 convertase and C5 convertase, as well as by degrading IgG. SCIN, originally found to be a prophage-encoded protein, inhibits all three complement pathways: the alternative, classical and lectin [6]. The SPIN protein prevents the oxidative burst by binding the enzyme myeloperoxidase, which is found inside neutrophil granules [39]. Moreover, the SPIN protein is responsible for binding convertase C3, preventing the release of the C3b subunit, which, in consequence, leads to inhibition of opsonization and phagocytosis. The SCIN protein, apart from its ability to bind convertase C3, is capable of binding the C3b subunit directly. In this way, all three complement pathways are blocked. ECB is another *S. aureus* protein responsible for complement inhibition. This protein binds to the C3d subunit, thereby blocking the alternative complement pathway by binding the H factor on the microbial surface. In the present study, all *S. aureus* strains isolated from nasal carriers harbored genes encoding ECB, which may facilitate balance maintenance between the host and colonizing strains [40]. SAK is another protein that is believed to play an important role in avoiding immune responses of the host. This protein reduces phagocytosis not only by binding the C3b subunit but also by degrading IgG through converting human plasminogen to serine protease plasmin bound to the bacterial surface [6]. Other proteins responsible for *S. aureus* immune evasion capacity are SBI and CHIPS. The latter one inhibits chemotaxis by binding the C5aR and FPRs, which results in an impaired response of neutrophils and monocytes [41]. SBI allows binding to immunoglobin IgG by the Fc domain, inhibiting phagocytosis [42,43]. The proteins interfere with complement activity through induction of C3 consumption by binding the C3dg fragment and C3a anaphylatoxin domain. This results in inhibition of the alternative pathway, as was shown by Burman et al. [44]. The regulation of SBI with serum components implies that this protein may be essential during early induction of inflammatory mediators following bacterial tissue invasion. The multifunction of SBI was confirmed by Smith et al. [42], who observed an increased survival of *S. aureus* exposed to human blood.

Having analyzed the mode of action of the selected proteins, we can conclude the *scn* gene is the most frequently detected (100%). This observation is in contrast to those revealed by Baptista et al. [10], Chai et al. [28] and Verkaik et al. [21], who confirmed that the *sak* and *chp* genes, isolated from food handlers’ hands (interdigital region, index finger, thumb and palm), nostrils, oral and nasal swabs were the most prevalent. In contrast to the above, Bano et al. [29] showed a similar prevalence of the *scn*, *sak* and *chp* genes in strains isolated from encompassing sputum, abscesses, aspirates, tissue samples, ear fluid, high vaginal swabs, throat swabs, pleural fluid, wound swabs, and skin lesions. Although *sak* and *chp* were not significantly prevalent in this study, the results seemed to be in line with those obtained by Baptista et al. [10], who confirmed the presence of 83.7% of *sak* and 67.4% of *chp* in strains isolated from food handlers’ hands (interdigital region, index finger, thumb and palm) and nostrils.

A completely different pathway for down-regulation of T cells and B cells is linked with enterotoxins such as SEA and SEP, which cause massive cytokine production and immunosuppression, followed by B cells apoptosis, resulting in toxic shock syndrome, through interactions with the T cell receptor and major histocompatibility complex II [11,45]. As revealed in this study, *sea* genes were the least frequently detected in tested strains (5.8%) and also in strains analyzed by Chai et al. [28] and Bano et al. [29]. Worth mentioning is the fact that products of *sep* genes, which were detected in 67.4% of the examined strains, may demonstrate more potent immunosuppressive features than the ones determined by *sea* genes.

In the present study, all examined strains were methicillin-susceptible. The percentage of MRSA strains isolated from invasive infections in Poland increased from 1999 to 2005 from 13% to 22%, and then decreased over 5 years, i.e., in the period between 2005 and 2020 from 24% to 13% [46]. MRSA is primarily associated with hospital environments in Poland, whereas MSSA strains are most frequently isolated from community patients. The increasing prevalence of MRSA strains detected in outpatients is also highlighted in the professional literature. These strains are considered to harbor multiple genes encoding virulence factors in contrast to HA-MRSA strains, which become multidrug-resistant at the cost of losing these genes [47]. It is noteworthy that community isolates of *S. aureus*, in particular MSSA strains, are statistically more likely to cause bacteremia in comparison to HA-MRSA strains [48]. It may be linked with the higher pathogenicity of MSSA than MRSA strains, which probably has something to do with the frequency of bacteremia in MSSA-infected patients. Bacteremia of *S. aureus* etiology has a 30-day infection-related mortality rate of 15–25% and an overall 90-day mortality rate of approximately 25–30% of patients [49,50]. Complications of bacteraemia can develop in one-third of patients and include infective endocarditis, bone infections and organ abscesses [51]. Risk factors for complications of bacteremia include nosocomial infection, long infection period, failure to identify the source of bacteremia and pneumonia as a cause of secondary bacteremia [52,53].

A significant decrease in mortality has been shown to be associated with improvements in the quality of care for patients with bacteremia of *S. aureus* etiology based on three key interventions: selection of an appropriate antibiotic, performance of echocardiography and specialist consultation by a physician prepared to consult patients with infections [54]. A decrease in mortality was shown when specialist consultations were provided to support management aimed at identifying the source of infection, implementing optimal antibiotic therapy, identification of complications and monitoring the effectiveness of treatment with follow-up blood cultures [55,56]. A 47% reduction in mortality was also demonstrated when specific activities were implemented targeting early identification and removal of the source of infection, the use of cloxacillin in treatment and the use of an appropriate treatment duration of no less than 14 days.

A PFGE analysis revealed the presence of 19 clonal types and five unique patterns. The high genetic diversity of the strains suggests a coincidental occurrence of the IEC types and IE genes in *S. aureus* isolates from outpatients, which confirms results obtained in previous studies by Masiuk et al. [3] and Kwiatkowski et al. [11]. No repetitive IEC distributions within all PFGE types was also confirmed. A comparative analysis of the frequency of the IEC cluster in humans and animals revealed that the number of IEC types detected in human isolates was much higher. It proves implementation of unique survival techniques allows for the avoidance of human immune system responses [10]. *scn* genes were found in all analyzed strains isolated from either cut wound infections or nasal swabs. Available data show that production of SCIN is determined by *scn* genes, present in each IEC type, and carried by the φSa3 prophage. Since IEC is considered a human adaptation marker, the genes are believed to be present mainly in human infection isolates of *S. aureus* and absent in strains isolated from animals reservoirs [57,58,59,60,61].

## 4. Materials and Methods

### 4.1. Bacterial Isolates

In the present study, 86 MSSA isolates belonging to the collection of the Department of Microbiology, Immunology and Laboratory Medicine of the Pomeranian Medical University in Szczecin (Poland) were analyzed. The strains were isolated from unrelated individuals: from nasal carriers (*n* = 43) with no SSTI episodes and from patients with cut wound infections (*n* = 43).

In all patients who demonstrated cut wound infections, simultaneous evaluation of the *S. aureus* nasal carrier state was performed. In all infected patients, the lack of *S. aureus* in nasal swabs was confirmed. In 32 of these patients, the infection was restricted to fingers; dorsal hand wound infection and palmary hand wound infection were confirmed in 9 and 2 patients, respectively. All patients selected for the study had resided in the West Pomeranian Voivodship for at least one year prior to material collection. All patients also denied hospitalization for a year and a half prior to material collection. Strain isolation and identification was carried out during routine microbiological diagnostics.

The study was approved by the Ethics Committee of the Pomeranian Medical University of Szczecin (approval numbers: KB-0012/04/01/14 and KB-0012/243/12/19/2).

### 4.2. DNA Isolation and PCR Assay

All bacterial strains were cultured at 37 °C for 24 h on Columbia agar with 5% sheep blood. After the incubation, a single colony was transferred into the tryptic soy broth (TSB, Merck Life Science, Poznan, Poland) and re-incubated under the same conditions. Cultures were centrifuged and the bacterial pellets were used for genomic DNA isolation using the Bacterial and Yeast Genomic DNA Purification Kit (EURx, Gdansk, Poland), according to the manufacturer’s protocol. In addition, a digestion procedure using lysostaphin (0.4 U/μL) (A&A Biotechnology, Gdansk, Poland) was included in the DNA isolation protocol.

Single PCR reactions for selected evasion genes (*sep*, *sak*, *sbi*, *spin*, *scn*, *chp* and *ecb*) were performed with designed primer pairs using the Primer Express 3.0 software (Applied Biosystems, Life Technologies, Carlsbad, CA, USA), based on *S. aureus* gene sequences available in the UniProt database (www.uniprot.org) (accessed on 12 December 2022). The *sea* gene was detected using the primer pairs described by Sanfilippo et al. [62]. The sequences of the oligonucleotide primers used in the current study are listed in Table 3.

PCR reactions were performed using the GoTaq Flexi DNA Polymerase Kit (Promega, Madison, WI, USA), dNTPs (A&A Biotechnology, Gdansk, Poland) and hot-start DNA polymerase (A&A Biotechnology, Gdansk, Poland). Gene amplification involved 35 cycles and included: initial activation temperature (95 °C for 30 s), annealing (60 °C for 30 s), extension (72 °C for 60 s) and final extension (72 °C for 10 min). PCR was performed using the Techne TC-512 96 Well Block Thermal Cycler (Keison Products, Chelmsford, UK). A 1.2% agarose gel with ethidium bromide (at a concentration of 0.1 µg/mL) was used for electrophoresis. Electrophoresis was carried out at 80 V for 60 min. Results were visualized under UV light using the GelDoc-It2 Imager system. *S. aureus* Newman (ATCC 25904) (harboring the *sea*, *sak*, *sbi*, *spin*, *scn*, *chp*, *ecb* genes) and *S. aureus* N315 (harboring the *sep* gene) strains were used as positive controls [63]. A sample without matrix DNA was used as the negative control.

### 4.3. PFGE Assay

Total bacterial DNA was isolated in accordance with the GenePath Group 6 Reagent Kit procedure, using CHEF Bacterial Genomic DNA Plug Kits (Bio-Rad, Hercules, CA, USA). *SmaI* restriction enzyme diluted in Tango buffer (ThermoScientific, Waltham, MA, USA) (1:10 ratio) was used for restriction digestion of DNA. CHEF DR III apparatus (Bio-Rad, Hercules, CA, USA) was used to perform PFGE in Tris-boran EDTA (TBE) buffer. The following conditions were used in the PFGE procedure: run time—20 h, initial switch time—2.2 s, final switch time—54.2 s, temperature—14 °C, voltage—6 V/cm, angel—120°. To visualize the results, the agarose gel was stained with 0.5 μg/mL ethidium bromide (Merck KGaA, Darmstatd, Germany) diluted in distilled water (1:10 ratio) for 20–30 min. Bacterial DNA of *S. aureus* ATCC 29213 strain was used as the normalizing standard on the gel. The results were read under UV light by using the GelDoc-It2 Imager system. FPQuest Software 4.5 (Bio-Rad, Hercules, CA, USA) was used to analyze restriction profiles. The obtained restriction patterns were classified using the Unweighted Pair Group Method with Arithmetic Mean (similarity coefficient [S_AB_] value = 63.0%) and the Dice coefficient (2.0%). Results are presented using a dendrogram.

### 4.4. Statistics

Data were analyzed using the GraphPad Prism 5.02 (GraphPad Software Inc., San Diego, CA, USA) software with the Chi-square test. *p* < 0.05 was adopted as statistically significant.

## 5. Conclusions

In conclusion, the diverse repertoire of genes determining invasion in the analyzed strains may prove the fact that strains isolated from the nasal cavity of healthy individuals and from outpatients with cut wound infections are much more advanced and adapted to evade the host immune response.

The most significant observation of the present study was the presence of genes encoding Ecb, conditioning the ability of *S. aureus* to suppress the host immune response associated with neutrophil activity. The presence of genes for Ecb production in the vast majority of strains isolated from healthy hosts may indicate a high degree of adaptability and ability to establish a balance between bacterium and host without triggering an immune response and, thus, inducing infection [64,65]. Ecb determines the ability of *S. aureus* to suppress the host immune response associated with neutrophil activity and, in addition, it has been shown to impair opso-dependent phagocytosis and, therefore, also affects immunological responses associated with complement activity. Strains colonizing patients but not causing infection and producing Ecb also have an inhibitory effect on the generation of immune response and, thus, the carrier state may also remain for a long time without affecting the antibody profile of the carrier [66].

Nevertheless, the pathogenic potential of strains demonstrating capacity to establish a carrier state without inducing an inflammatory state cannot be excluded. Therefore, the detailed genetic analysis, including detection of the single virulence genes within *S. aures* populations isolated from particular infections or carrier states, is a foreground for us to plan more extensive studies on specific staphylococcal populations through the use of methods of sequencing their genomes.

In order to determine the presence of genes encoding other virulence factors, such as enzymes or exotoxins, and to find the possible relationship between their presence and the clinical picture of particular SSTIs, further studies are required. Moreover, in order to fully understand the mechanism underlying the persistence of a carrier state without causing symptoms of SSTIs, as well as determine the clinical picture and final outcome of particular SSTIs, comparisons of the genotypic characteristics of *S. aureus* with the antibody profiles of each colonized individual are in our immediate plans.

## Figures and Tables

**Table 1:** The number and prevalence of selected immune evasion (IE) genes in methicillin-susceptible *S. aureus* strains isolated from nasal carriers and from outpatients with cut wound infections.

IE Gene	Result	Total (*n* = 86) *n* (%)	Cut Wounds (*n* = 43) *n* (%)	Nasal Carriers (*n* = 43) *n* (%)	*p*-Value
*sea*	Positive	5 (5.81)	3 (6.98)	2 (4.65)	0.6449
	Negative	81 (94.19)	40 (93.02)	41 (95.35)	
*sak*	Positive	72 (83.72)	39 (90.7)	33 (76.74)	0.0797
	Negative	14 (6.28)	4 (9.3)	10 (23.26)	
*chp*	Positive	58 (67.44)	29 (67.44)	29 (67.44)	1.0
	Negative	28 (32.56)	14 (32.56)	14 (32.56)	
*scn*	Positive	86 (100.0)	43 (100.0)	43 (100.0)	1.0
	Negative	0 (0.0)	0 (0.0)	0 (0.0)	
*sep*	Positive	58 (67.44)	26 (60.47)	32 (74.42)	0.1674
	Negative	28 (32.56)	17 (39.53)	11 (25.58)	
*ecb*	Positive	79 (91.86)	36 (83.72)	43 (100.0)	0.0058
	Negative	7 (8.14)	7 (16.28)	0 (0.0)	
*sbi*	Positive	82 (95.35)	42 (97.67)	40 (93.02)	0.3058
	Negative	4 (4.65)	1 (2.33)	3 (6.98)	
*spin*	Positive	77 (89.53)	38 (88.37)	39 (90.7)	0.7246
	Negative	9 (10.47)	5 (11.63)	4 (9.3)	

**Table 2:** The number and prevalence of immune evasion cluster (IEC) types in methicillin-susceptible *S. aureus* strains isolated from nasal carriers and from outpatients with cut wound infections.

IEC Type	Result	Total (*n* = 86) *n* (%)	Cut Wounds (*n* = 43) *n* (%)	Nasal Carriers (*n* = 43) *n* (%)	*p*-Value
A (*sea*-*sak*-*chp*-*scn*)	Positive	0 (0.0)	0 (0.0)	0 (0.0)	0.0
	Negative	86 (100.0)	43 (100.0)	43 (100.0)	
B (*sak*-*chp*-*scn*)	Positive	1 (1.16)	1 (2.33)	0 (0.00)	0.3145
	Negative	85 (98.84)	42 (97.67)	43 (100.0)	
C (*chp*-*scn*)	Positive	3 (3.49)	3 (6.98)	0 (0.00)	0.0779
	Negative	83 (96.51)	40 (93.02)	43 (100.0)	
D (*sea*-*sak*-*scn*)	Positive	1 (1.16)	1 (2.33)	0 (0.00)	0.3145
	Negative	85 (98.84)	42 (97.67)	43 (100.0)	
E (*sak*-*scn*)	Positive	21 (24.42)	11 (25.58)	10 (23.26)	0.8018
	Negative	65 (75.58)	32 (74.42)	33 (76.74)	
F (*sep*-*sak*-*chp*-*scn*)	Positive	43 (50.0)	23 (53.49)	20 (46.51)	0.5176
	Negative	43 (50.0)	20 (46.51)	23 (53.49)	
G (*sep*-*sak*-*scn*)	Positive	2 (2.33)	1 (2.33)	1 (2.33)	1.0
	Negative	83 (96.51)	42 (97.67)	42 (97.67)	
H (*scn*)	Positive	2 (2.33)	1 (2.33)	1 (2.33)	1.0
	Negative	83 (96.51)	42 (97.67)	42 (97.67)	
Non-typeable (all IEC genes detected)	Positive	13 (15.12)	2 (4.65)	11 (25.58)	0.0067
	Negative	73 (84.88)	41 (95.35)	32 (74.42)	

**Table 3:** Oligonucleotide sequences used to detect selected immune evasion genes in *S. aureus* strains.

Gene	Sequence (5′→3′)	Size (bp)	Reference
*sak*	F: TGC GAC AGC ATA TAA AGA GTT TAG AGT A R: TCT GGG ACA ACA AAA CCT TTT TC	137	This study
*sbi*	F: GAA GAA CAA CGT AAC CAA TAC ATC AAA R: GGG TTC TTG CTG TCT TTA AGT GAT T	94	This study
*sea*	F: CAA ATA AAT CGT AAT TAA CCG AAG GTT C R: GAA AAA AGT CTG AAT TGC AGG GAA CA	560	[62]
*spin*	F: CAT GTA GTA CGA GTC CAT TTT GAG AAT AA R: GCT ACG GCA ATG GTA GGT GTT T	96	This study
*chp*	F: GCG AAA GCT TTT ACT TTT GAA CC R: CCT AGC GTT GTA GGA AGA CCA	125	This study
*sep*	F: CCG CCA TAC ATA CAA GCT GTT R: GTT CAA AAG ACA CCG CCA AT	115	This study
*scn*	F: ATT CAT TCG ATG TTG GCA AG R: ACT TGC GGG AAC TTT AGC AA	101	This study
*ecb*	F: ACT AGA TCG ATT TGT CTT TGT AAT TTT R: GTT GCA ACA CAC CGT AAA GC	120	This study

F—forward; R—reverse; bp—base pair.

## Data Availability

Data are contained within the article.

## Supplementary Materials

The following supporting information can be downloaded at: https://www.mdpi.com/article/10.3390/antibiotics13080730/s1. Figure S1: A heat-map showing the comparison of the eighty six methicillin-susceptible Staphylococcus aureus strains isolated from nasal carriers and from outpatients with cut wound infections; Table S1: Detailed summary of results on presence of selected immune evasion (IE) genes, immune evasion cluster (IEC) types, and clonal diversity in methicillin-susceptible Staphylococcus aureus isolated from nasal carriers and from outpatients with cut wound infections.
