# Supplementary material for: Prevalence of Selected Immune Evasion Genes and Clonal Diversity in Methicillin-Susceptible Staphylococcus aureus Isolated from Nasal Carriers and Outpatients with Cut Wound Infections
Source: Antibiotics (Basel). 2024 Aug 3;13(8):730. doi: 10.3390/antibiotics13080730 (PMC11350705; doi:10.3390/antibiotics13080730)

Supplementary Table S1. Detailed summary of results on presence of selected immune evasion (IE) genes, immune evasion cluster (IEC) types, and clonal diversity in methicillin-susceptible *Staphylococcus aureus* isolated from nasal carriers and from outpatients with cut wound infections.

Strain number	Source	IEC genes						IEC type	Other IE genes		PFGE type
		sea	*sak*	*chp*	*scn*	*ecb*	*sep*		*sbi*	*spin*	
1	Cut wound (FI)	-	-	+	+	-	-	C	+	+	F
2	Cut wound (DH)	+	+	-	+	-	-	D	+	+	F
3	Cut wound (FI)	-	-	+	+	-	-	C	+	-	K
4	Cut wound (FI)	-	+	-	+	-	+	G	+	-	H
5	Cut wound (FI)	-	-	+	+	-	-	C	+	+	H
6	Cut wound (FI)	-	+	-	+	-	-	E	+	-	H
7	Cut wound (FI)	-	+	+	+	-	-	B	+	-	I
8	Cut wound (DH)	-	+	+	+	+	+	F	+	+	I
9	Cut wound (FI)	-	+	+	+	+	+	F	+	+	Q
10	Cut wound (FI)	-	+	+	+	+	+	F	+	+	K
11	Cut wound (FI)	-	+	+	+	+	+	F	+	+	P
12	Cut wound (DH)	-	+	+	+	+	+	F	+	+	D
13	Cut wound (FI)	-	+	+	+	+	+	F	+	+	A
14	Cut wound (FI)	+	+	+	+	+	+	-	+	+	P
15	Cut wound (FI)	-	+	+	+	+	+	F	+	+	N
16	Cut wound (FI)	+	+	+	+	+	+	-	+	+	Un3
17	Cut wound (FI)	-	+	+	+	+	+	F	+	+	L
18	Cut wound (FI)	-	+	+	+	+	+	F	+	+	D
19	Cut wound (FI)	-	+	+	+	+	+	F	+	+	K
20	Cut wound (FI)	-	-	-	+	+	-	H	-	-	L
21	Cut wound (DH)	-	+	-	+	+	-	E	+	+	S
22	Cut wound (FI)	-	+	-	+	+	-	E	+	+	S
23	Cut wound (FI)	-	+	-	+	+	-	E	+	+	S
24	Cut wound (DH)	-	+	-	+	+	-	E	+	+	C
25	Cut wound (DH)	-	+	-	+	+	-	E	+	+	H
26	Cut wound (FI)	-	+	-	+	+	-	E	+	+	Un1
27	Cut wound (FI)	-	+	-	+	+	-	E	+	+	N
28	Cut wound (FI)	-	+	-	+	+	-	E	+	+	E
29	Cut wound (FI)	-	+	-	+	+	-	E	+	+	F
30	Cut wound (FI)	-	+	-	+	+	-	E	+	+	Un4
31	Cut wound (FI)	-	+	+	+	+	+	F	+	+	H
32	Cut wound (PH)	-	+	+	+	+	+	F	+	+	A
33	Cut wound (DH)	-	+	+	+	+	+	F	+	+	L
34	Cut wound (FI)	-	+	+	+	+	+	F	+	+	C
35	Cut wound (DH)	-	+	+	+	+	+	F	+	+	O
36	Cut wound (FI)	-	+	+	+	+	+	F	+	+	S
37	Cut wound (PH)	-	+	+	+	+	+	F	+	+	G
38	Cut wound (FI)	-	+	+	+	+	+	F	+	+	Un2
39	Cut wound (DH)	-	+	+	+	+	+	F	+	+	K
40	Cut wound (FI)	-	+	+	+	+	+	F	+	+	J
41	Cut wound (FI)	-	+	+	+	+	+	F	+	+	R
42	Cut wound (FI)	-	+	+	+	+	+	F	+	+	K
43	Cut wound (FI)	-	+	+	+	+	+	F	+	+	A
44	Nasal swab	-	-	-	+	+	-	H	-	-	O
45		-	-	-	+	+	+	-	-	-	L
46		-	-	+	+	+	+	-	+	+	D
47		-	-	+	+	+	+	-	+	+	G
48		-	-	+	+	+	+	-	+	+	L

49	-	-	+	+	+	+	-	-	-	A
50	-	-	+	+	+	+	-	+	-	C
51	-	+	+	+	+	+	F	+	+	E
52	-	+	+	+	+	+	F	+	+	R
53	+	+	+	+	+	+	-	+	+	H
54	-	+	+	+	+	+	F	+	+	K
55	-	+	+	+	+	+	F	+	+	A
56	-	+	+	+	+	+	F	+	+	P
57	-	+	+	+	+	+	F	+	+	K
58	+	+	+	+	+	+	-	+	+	H
59	-	+	+	+	+	+	F	+	+	K
60	-	+	+	+	+	+	F	+	+	K
61	-	+	+	+	+	+	F	+	+	R
62	-	+	+	+	+	+	F	+	+	B
63	-	+	+	+	+	+	F	+	+	J
64	-	+	-	+	+	-	E	+	+	E
65	-	+	-	+	+	-	E	+	+	P
66	-	+	-	+	+	-	E	+	+	K
67	-	+	-	+	+	-	E	+	+	M
68	-	+	-	+	+	-	E	+	+	M
69	-	+	-	+	+	-	E	+	+	E
70	-	+	-	+	+	-	E	+	+	K
71	-	+	-	+	+	-	E	+	+	M
72	-	+	-	+	+	-	E	+	+	A
73	-	+	-	+	+	-	E	+	+	E
74	-	+	+	+	+	+	F	+	+	B
75	-	+	+	+	+	+	F	+	+	Un5
76	-	+	+	+	+	+	F	+	+	K
77	-	+	+	+	+	+	F	+	+	D
78	-	+	-	+	+	+	G	+	+	Q
79	-	-	-	+	+	+	-	+	+	D
80	-	+	+	+	+	+	F	+	+	M
81	-	+	+	+	+	+	F	+	+	D
82	-	-	+	+	+	+	-	+	+	K
83	-	+	+	+	+	+	F	+	+	C
84	-	-	+	+	+	+	-	+	+	F
85	-	+	+	+	+	+	F	+	+	D
86	-	+	+	+	+	+	F	+	+	A

Legend: „+" – positive; „-" – negative; IEC - immune evasion cluster; IE - immune evasion, Un – uniqe strain, DH – dorsal hand wound infection; FI – finger infection; PH – palmary hand wound infection.

Supplementary Figure S1. A heat-map showing the comparison of the eighty six methicillin-susceptible *Staphylococcus aureus* strains isolated from nasal carriers and from outpatients with cut wound infections.

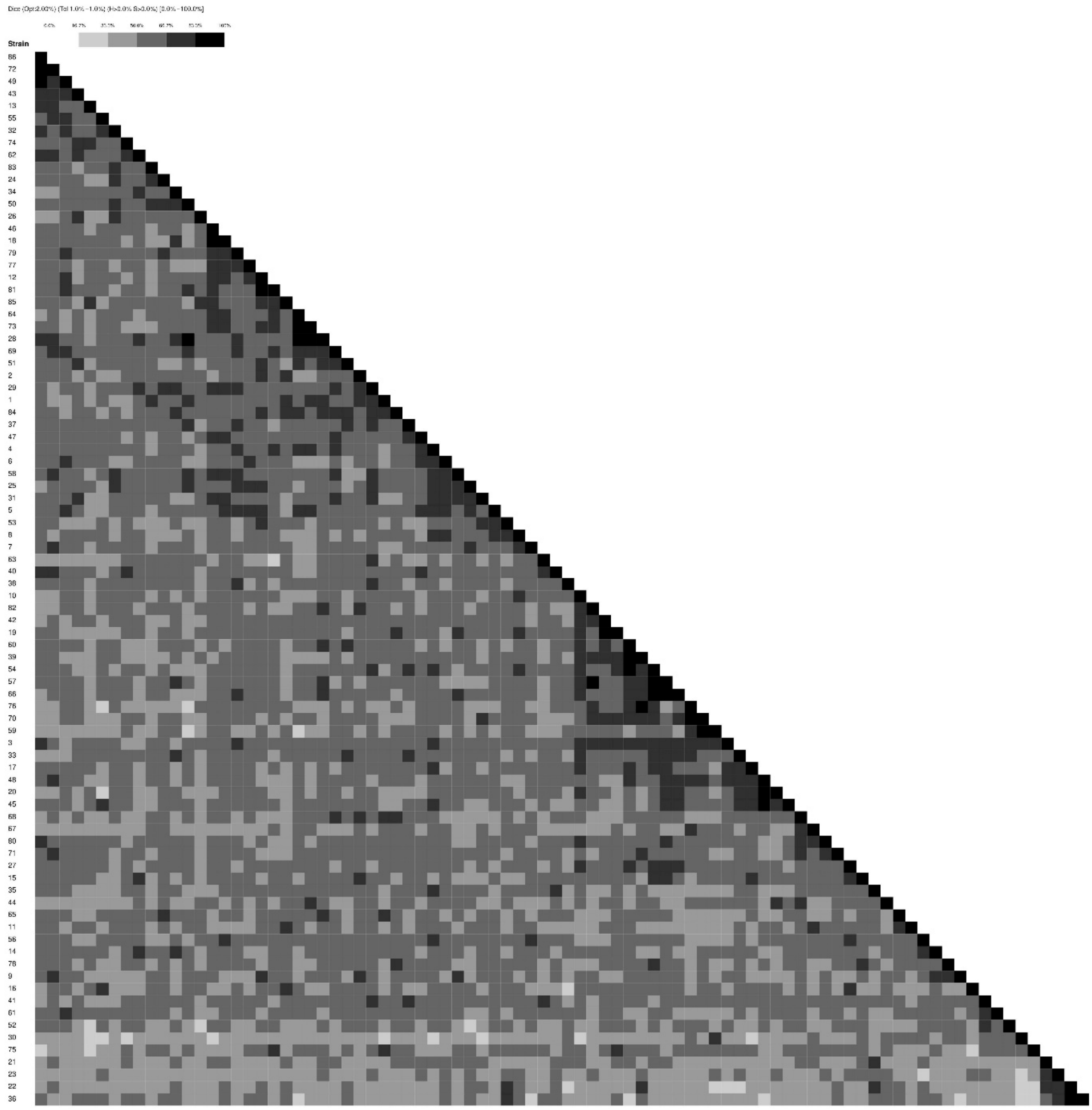

Supplement: Supplementary file 1 [file antibiotics-13-00730-s001.zip › antibiotics-3097609-supplementary.pdf]
